# Anxiety and vital signs in cancer patients and music students before and after a music intervention: a quasi-experimental pilot study*

**DOI:** 10.1590/1518-8345.7834.4635

**Published:** 2025-11-03

**Authors:** Raquel Caballero-de-la-Calle, Patricia Ferrero-Sereno, Miguel Ángel García-Garrido, Ana Ruiz-Rodríguez, María Nieves Moro-Tejedor, Miriam Leñero-Cirujano

**Affiliations:** 1 Universidad Alfonso X el Sabio, Facultad de Ciencias Biomédicas y de la Salud, Grado de Enfermería, Madrid, Spain.; 2 Universidad Alfonso X el Sabio, Facultad de Ciencias Escénicas, Grado de Interpretación Musical, Madrid, Spain.; 3 Hospital General Universitario Gregorio Marañón, Madrid, Spain.; 4 Instituto de Investigación Sanitaria Gregorio Marañón, Madrid, Spain.; 5 Universidad Complutense de Madrid, Facultad de Enfermería, Fisioterapia y Podología, Departamento de Enfermería, Madrid, Spain.

**Keywords:** Integrative Oncology, Holistic Nursing, Interdisciplinary Placement, Music, Anxiety, Vital Signs

## Abstract

to analyze the level of anxiety and variation in the vital signs of cancer patients and performers (music students) before and after a live musical intervention.

quasi-experimental pilot study with no control group. An interdisciplinary musical intervention was carried out between Nursing and Music Performance students. The level of anxiety was assessed with the Hamilton scale and the variation in vital signs before and after the intervention in a day hospital in cancer patients and in interpreters, in the latter the variables were also analyzed before and after the intervention. The Wilcoxon test was used to analyze the data.

18 patients and 10 music students took part. Variations in vital signs were observed in both groups, as well as a decrease in the level of anxiety in the patients at the post-intervention moment. In the students, the level of anxiety was higher at the beginning of the dress rehearsal compared to the rest of the evaluated moments at the hospital.

live music interventions could improve anxiety levels in both cancer patients and performers.

## Highlights

**(1)** Live music interventions can improve anxiety levels. **(2)** The impact of live music is beneficial for patients and performers. **(3)** Interdisciplinary intervention between students in real health contexts. **(4)** These interventions promote collaborative work and active learning.

## Introduction

The diagnosis and treatment of cancer has a profound negative impact on the well-being and quality of life of those who suffer from it. Cancer patients often manifest feelings of anxiety, anger, fear and sadness, which are exacerbated by the clinical symptoms of the disease (nausea, vomiting, anorexia, fatigue, insomnia, pain, among others). This combination of factors can generate a continuous feeling of malaise^(1)^.

In this sense, interventions aimed at improving the well-being and comfort of these patients are fundamental^(2-3)^. A report published by the World Health Organization (WHO) highlighted the existence of numerous studies highlighting the importance of the arts in health^(4)^. In this context, the interpretation of live music is proposed as a holistic and complementary resource to conventional cancer treatment, capable of reducing some of the symptoms associated with the process, increasing the quality of life of these patients and improving the therapeutic relationship^(5-9)^. In addition, music therapy is recognized as a nursing intervention in the Nursing Interventions Classification (NIC), defining it as the “use of music to help achieve a specific, physiological, behavioral and/or feeling change”^(10)^. These musical interventions not only have a positive impact on patients, caregivers and healthcare professionals, but also affect the performers, creating an enriching synergy between music and care. Performing live music in hospital environments represents a challenge for the latter, who must act with special sensitivity and empathy in order to adapt to a different audience from the one they normally address in traditional concert halls^(11)^. There are few studies that consider this triangular approach of patients-healthcare professionals-interpreters when evaluating the effect of music in healthcare environments^(11)^.

In this study, these three strands (patients-healthcare professionals-interpreters) take center stage, developing within the framework of a collaborative and interdisciplinary challenge between two degrees at the Universidad Alfonso X El Sabio: Nursing Degree and Music Performance Degree. The main aim of the research was to assess the level of anxiety and variation in the vital signs of cancer patients and interpreters (music students) before and after a live musical intervention.

## Method

### Type of study

Quasi-experimental pilot study with no control group and no randomization, evaluating the effect of a live music intervention on anxiety levels and vital signs in cancer patients and music students (interpreters of the intervention). In the latter, these variables were also assessed before and after the general trial. The guidelines of the TIDier (Template for Intervention Description and Replication)^(12)^ checklist were followed for the development of this study.

It is hypothesized that the performance of live music in a hospital setting can influence the well-being of both populations. In cancer patients, it is expected that anxiety levels will decrease after the musical intervention. In music students, anxiety levels are expected to be higher in the live music intervention than in previous rehearsals.

### Setting and period of the study

The intervention was carried out in the medical oncology day hospital unit of the Rey Juan Carlos University Hospital (HURJC) in Madrid, Spain. The previous trials were carried out at the Alfonso X El Sabio University (UAX) in Villanueva de la Cañada, Spain. This research was carried out during the months of February to May 2024, following the academic calendar.

### Participants

The patient population was made up of those who attended the HURJC day hospital to receive their chemotherapy cycle on the days selected for the live musical performances. On average, the hospital receives approximately 15 patients a day for chemotherapy treatment. The inclusion criteria were being of legal age and voluntarily agreeing to take part in the study. The exclusion criteria were patients diagnosed with dementia or cognitive impairment of any degree, patients with language disorders of any etiology that prevent communication and comprehension, and patients who had already participated in previous live music sessions in our study.

The interpreter population consisted of all music students enrolled in the Music Interpretation and Modern Music Interpretation courses (N=10) at UAX in the 2023-2024 academic year, who voluntarily agreed to take part in the study. They were invited to take part during a brief information session at the end of one of their usual classes.

As this was a pilot study, sampling was opportunistic, consecutive to the entire accessible population, taking into account the inclusion and exclusion criteria.

### Intervention by live musical interpretation

An interdisciplinary collaborative educational activity was planned between students from the Music Performance Courses and the UAX Nursing Course.

Two months before the intervention, the music students held their rehearsals on the university premises. A week before the performance at the hospital, a rehearsal was held, during which the first measurements were taken on the music students.

The musical intervention at the hospital consisted of live performances by soloists or duos of musical pieces previously selected by the music students and their teachers, using instruments such as piano, violin and guitar. The criteria for selecting the pieces included their ability to promote relaxation and the patients’ emotional well-being: Chopin’s “Scherzo No. 4 Op. 54” for solo piano, Bizet’s ‘Habanera’ and Falla’s “La vida breve” for piano four hands, Schnittke’s “Suite in the Old Style” and Skoryk’s “Spanish Dance” for violin and piano duos, and jazz standards for keyboard and electric guitar.

These pieces were performed in 30-minute sessions in the oncology day hospital while the patients were receiving their chemotherapy treatment. The days on which the sessions took place were kept anonymous for the patients, except for the hospital management and the unit’s nursing staff, for logistical reasons. Two musical interventions were carried out in one day, repeating the same procedure for a total of three days in order to capture a larger sample. The scheduling of the intervention in the hospital was done in conjunction with the nursing management of the institution. Patients in the day hospital received only one music session during their treatment.

### Study variables and data collection

Sociodemographic variables (age, gender, marital status) and clinical variables (blood pressure, heart rate, oxygen saturation and degree of anxiety) were collected from both populations. Vital signs were measured using calibrated blood pressure monitors and pulse oximeters. The degree of anxiety was assessed using the Hamilton Anxiety Scale (HAS)^(13)^. The HAS scale is a 14-item heteroapplied scale, 13 of which refer to anxious signs and symptoms (anxious mood, tension, fears, insomnia, intellectual/cognitive, depressed mood, general somatic [muscular] symptoms, general somatic [sensory] symptoms, cardiovascular symptoms, respiratory symptoms, gastrointestinal symptoms, genitourinary symptoms and autonomic symptoms) and the last one assesses the interviewee’s behavior, with good internal consistency (Cronbach’s α: 0.88). Each item is scored from 0 (absent) to 4 (disabling) points. There are no cut-off points: the higher the score on the scale, the greater the intensity.

In the patient group, other clinical variables related to their pathology were also collected, such as tumor type, stage, current treatment and pain level (assessed using the visual analog scale). In the student group, a final evaluation of the experience was recorded (very good/good/regular/bad/very bad) and their intention to possibly repeat the intervention (yes/no).

Sociodemographic and clinical variables were recorded both before and after the musical intervention and, in the case of the students, they were also collected before and after the general test. To avoid bias in the responses, participants were not reminded of the information previously provided. In addition, the identities of the participants were coded to guarantee the anonymization of the data.

Data collection was carried out by volunteer students from the 2nd year of the Nursing course. Prior to collection, a training session was scheduled with the students to inform them about the nature of the study and instruct them on how to properly collect the variables, following standardized techniques to ensure the accuracy and consistency of the clinical data.

### Data analysis

Quantitative variables were presented with means and standard deviations or medians and interquartile ranges, depending on their distribution. Qualitative variables were expressed as percentages and frequencies. Wilcoxon’s test was used to compare the paired samples before and after the musical intervention, as well as the test, which is suitable for small samples that do not follow a normal distribution. The data was analyzed using the statistical program SPSS v.29^(14)^. A value of p<0.05 was considered for statistical significance.

### Ethical aspects

The study was carried out in accordance with the Declaration of Helsinki, compliance with the Biomedical Research Act and compliance with the standards of Good Clinical Practice insofar as they are applicable, which include the monitoring of study participants to ensure the quality of the data and the protection of participating subjects. Written approval was obtained from the ethics committee of both institutions involved in the study.

Patients and students were informed about the aim and method of the research and written informed consent was obtained. Participation was anonymous and voluntary, with the participant having the right to withdraw at any time. To preserve the confidentiality of the data, an alphanumeric code was assigned to each participant. Personal data was processed in accordance with General Regulation (EU) 2016/679 on data protection and Organic Law 3/2018 on data protection and the guarantee of digital rights. This study was funded by the XVI UAX-FUAX 2023 Call for Grants for the Development of Research Projects.

## Results

Initially, the sample consisted of 23 patients, 5 of whom did not complete the study due to early termination of their treatment before the musical intervention. The sample therefore consisted of 18 patients, equal numbers of men and women, aged between 34 and 84, with an average age of 57.9 (16.3) years. In the case of the students, the sample was made up of all the students enrolled in the music course (N=10) who joined voluntarily, with a participation rate of 100%. The students had an average age of 22 (1.1) years, ranging from 19 to 22 years, of which 70% were women (Table 1).

**Table 1-:** Descriptive statistics: sociodemographic and clinical variables of patients (N* = 18) and students (N* = 10). Madrid, Spain, 2024

Variables	Cancer patients N* (%) ^†^	Music students N* (%) ^†^
**Gender**		
Women	9 (50%)	7 (70%)
Men	9 (50%)	3 (30%)
**Marital status**		
Married	12 (66.7%)	0
Divorced	1 (5.6%)	0
Single	4 (22.2%)	10 (100%)
Widowed	1 (5.6%)	0
**Tumor origin**		
Digestive	5 (27.8%)	--
Respiratory	7 (38.9%)	--
Gynecological	5 (27.7%)	--
Blood	1 (5.6%)	--
**Tumor stage**		
I	2 (11.1%)	--
II	0	--
III	9 (50%)	--
IV	7 (38.9%)	--
**Type of chemotherapy treatment**		
Neoadjuvant	8 (44.4%)	--
Adjuvant	2 (11.1%)	--
Palliative	8 (44.4%)	--

*N = Number of participants; ^†^Percentage

Table 2 shows the descriptive statistics for vital parameters before and after the intervention. In the group of patients, there was an increase in blood pressure values, 12.5 mmHg in the systolic (p=0.013) and 2.0 mmHg in the diastolic (p=0.214), and in heart rate (2.5 bpm; p=0.038) in the post-intervention compared to the moment before the musical intervention. No changes were observed in the level of pain. In the student group, the behavior of the vital constants was very similar at the moments before and after the rehearsal, as well as the intervention in the hospital, with significant differences being observed in systolic blood pressure before and after the rehearsal, with a post-rehearsal decrease of 16.5 mmHg compared to the moment before the rehearsal (p=0.028). There were no significant differences in the other vital parameters (p>0.05) or between the moments before and after the rehearsal compared to the moments before and after the musical intervention (p>0.05).

**Table 2-:** Distribution of mean vital signs in patients (N* = 18) and music students (N* = 10) before and after musical rehearsal (students) and before and after musical intervention in hospital. Madrid, Spain, 2024

	**Cancer patients**			**Music students**					
	**Hospital intervention**			**Music rehearsal**			**Hospital intervention**		
**Variables**	**Before**	**After**	**p value** ^§^	**Before**	**After**	**p value** ^§^	**Before**	**After**	**p value** ^§^
	Md ^†^ [RIQ] ^‡^	Md ^†^ [RIQ] ^‡^		Md ^†^ [RIQ] ^‡^	Md ^†^ [RIQ] ^‡^		Md ^†^ [RIQ] ^‡^	Md ^†^ [RIQ] ^‡^	
Systolic blood pressure	116.0	128.5	**0.013**	126.5	110.0	**0.028**	116.0	114.5	0.260
	[101.0-133.5]	[107.3-137.3]		[117.5-130.0]	[102.8-122.5]		[111.0-127.0]	[101.8-119.8]	
Diastolic blood pressure	71.5	73.5	0.214	75.0	73.0	0.779	76.0	73.5	0.359
	[59.5-78.5]	[64.0-82.3]		[65.0-82.5]	[70.0-77.0]		[67.3-86.3]	[66.5-79.5]	
Heart rate	73.5	76.0	**0.038**	82.5	89.0	0.306	87.00	93.5	0.386
	[66.8-76.0]	[65.8-80.3]		[72.8-93.0]	[68.8-101.0]		[79.0-98.8]	[80.5-102.0]	
O _2_ saturation	97.0	97.0	0.591	98.0	98.0	0.491	97.0	97.0	0.334
	[95.0-98.3]	[94.5-99.3]		[96.8-99.0]	[95.8-98.3]		[94.8-99.3]	[94.8-98.3]	
Level of pain	1.0	1.0	0.180	--	--	--	--	--	--
	[1.0-2.3]	[1.0-2.3]							

*N = Number of participants; ^†^Md = Median; ^‡^[RIQ] = Interquartile range; ^§^p-value of Wilcoxon test

Table 3 shows the distribution of the median anxiety scores. In the patient sample, in general, the scores on the items decreased post-intervention compared to pre-intervention, with no significant differences, except for the fear item (p=0.034) and general somatic (muscular) symptoms (p=0.013). In the total score of the scale, there was a decrease of 4 points post-intervention (p=0.004).

**Table 3-:** Distribution of median anxiety scores in patients (N* = 18) and music students (N* = 10) in the pre and post musical rehearsal (students) and in the pre and post musical intervention in the hospital. Madrid, Spain, 2024

	**Cancer patients**			**Music students**					
	**Hospital intervention**			**Musical rehearsal**			**Hospital intervention**		
**Hamilton Scale items**	**Before**	**After**	**p value** ^§^	**Before**	**After**	**p valor** ^§^	**Before**	**After**	**p value** ^§^
	Md ^†^ [RIQ] ^‡^	Md ^†^ [RIQ] ^‡^		Md ^†^ [RIQ] ^‡^	Md ^†^ [RIQ] ^‡^		Md ^†^ [RIQ] ^‡^	Md ^†^ [RIQ] ^‡^	
Anxious	1.0	0.0	0.083	2.0	0.0	**0.038**	0.0	0.0	0.317
	[0.0-2.0]	[0.0-1.0]		[0.0-2.0]	[0.0-1.3]		[0.0-1.0]	[0.0-0.3]	
Tension	0.5	0.0	0.477	0.0	0.0	0.414	0.0	0.0	1
	[0.0-1.3]	[0.0-1.0]		[0.0-2.0]	[0.0-1.3]		[0.0-1.0]	[0.0-1.0]	
Fear	0.5	0.0	**0.034**	1.0	0.0	0.058	0.0	0.0	0.317
	[0.0-1.0]	[0.0-1.0]		[0.8-1.3]	[0.0-0.5]		[0.0-0.0]	[0.0-0.0]	
Insomnia	1.0	1.0	0.102	1.0	0.0	**0.024**	0.0	0.0	1
	[0.0-2.0]	[0.0-1.3]		[0.0-2.0]	[0.0-0.3]		[0.0-0.3]	[0.0-0.3]	
Intellectual (cognitive)	0.0	0.0	0.305	0.0	0.0	0.564	0.0	0.0	1
	[0.0-2.0]	[0.0-1.0]		[0.0-2.3]	[0.0-2.0]		[0.0-0.3]	[0.0-0.3]	
Depressed	0.0	0.0	0.058	1.0	0.0	**0.038**	0.0	0.0	1
	[0.0-1.3]	[0.0-1.0]		[0.0-2.0]	[0.0-0.0]		[0.0-0.0]	[0.0-0.0]	
Somatic muscle symptoms	1.0	0.0	**0.013**	1.5	1.0	0.119	0.0	0.0	0.317
	[0.0-2.0]	[0.0-1.0]		[0.0-2.3]	[0.0-1.3]		[0.0-1.0]	[0.0-0.3]	
Somatic sensory symptoms	1.0	0.0	0.059	0.5	0.0	0.102	0.0	0.0	0.317
	[0.0-1.0]	[0.0-1.0]		[0.0-1.5]	[0.0-1.3]		[0.0-0.0]	[0.0-0.3]	
Cardiovascular symptoms	0.0	0.0	0.248	0.5	0.0	0.157	0.0	0.0	0.083
	[0.0-1.0]	[0.0-0.3]		[0.0-2.0]	[0.0-2.0]		[0.0-0.3]	[0.0-1.0]	
Respiratory symptoms	0.0	0.0	0.238	0.0	0.0	1	0.0	0.0	0.317
	[0.0-1.0]	[0.0-0.3]		[0.0-0.5]	[0.0-1.0]		[0.0-0.3]	[0.0-0.0]	
Gastrointestinal symptoms	1.0	0.0	0.405	0.0	0.0	0.157	0.0	0.0	1
	[0.0-1.3]	[0.0-2.0]		[0.0-1.3]	[0.0-1.0]		[0.0-0.3]	[0.0-0.3]	
Genitourinary symptoms	0.0	0.0	0.058	0.0	0.0	1	0.0	0.0	1
	[0.0-1.0]	[0.0-0.0]		[0.0-0.0]	[0.0-0.0]		[0.0-0.0]	[0.0-0.0]	
Autonomic symptoms	1.0 [0.0-1.3]	0.0	0.058	1.0	0.0	0.655	0.0	0.5	0.564
		[0.0-1.0]		[0.0-1.0]	[0.0-2.0]		[0.0-1.3]	[0.0-1.0]	
Interview behavior	1.0	0.5	0.739	1.5	1.0	0.488	0.50	1.0	0.059
	[0.0-1.0]	[0.0-1.0]		[0.0-2.0]	[0.0-2.0]		[0.00-1.00]	[0.0-2.0]	
Hamilton Scale total	9.5	5.5	**0.004**	11.5	5.0	**0.019**	2.5	4.0	0.269
	[8.0-15.3]	[3.0-9.3]		[5.8-18.8]	[0.7-14.0]		[0.0-7.8]	[0.0-7.3]	

*N = Number of participants; ^†^Md = Median; ^‡^[IQR] = Interquartile Range; ^§^p-value Wilcoxon test

In the music student population, at the time of the dress rehearsal, there was a decrease in most of the scale items at the end of the interpretation compared to the beginning of the rehearsal, with significant differences in the items: anxious mood (p=0.038), insomnia (p=0.024) and depressed mood (p=0.038). However, at the time of the musical intervention in the hospital, the scores of the items were very similar to each other, with no statistically significant differences (p>0.005).

Nor were there any differences between the two moments after the rehearsal and the intervention in the hospital (p>0.005), but between the two previous moments in both environments, specifically in the items: anxious mood (p=0.024), fear (p=0.008), depressed mood (p=0.038), muscular somatic symptoms (p=0.031), sensory somatic symptoms (p=0.038), behavior in the interview (p=0.034) and in the total score of the scale (p=0.012). The latter showed that anxiety levels were significantly higher at the beginning of the dress rehearsal (11.5; p=0.019) compared to the other measurement moments, both after the rehearsal (5.0; p=0.019) and before and after the musical intervention (2.50 and 4.0, respectively; p=0.012). The degree of anxiety was slightly higher at the end of the intervention than at the beginning, although this difference was not statistically significant (p=0.269) (Table 3).

As for the evaluation of the experience, all the students rated it as very good (80%) and good (20%), saying that they would all be willing to take part again if the intervention were repeated in subsequent courses.

## Discussion

This study describes the results of the implementation of an interdisciplinary innovation educational intervention, which integrates the arts and health sciences, in the context of biopsychosocial care and teamwork for students in a real health environment. The results offer an interesting insight into the role of music in physical, psychological and emotional well-being, highlighting its potential as a complementary tool in the health field.

The significant increase in systolic blood pressure and heart rate in the patients after the intervention, compared to before, could be due to a physiological response to the musical stimulus. However, this variation in vital constants cannot be attributed exclusively to the intervention, as it may be influenced by the administration of chemotherapy treatment, among other factors. The literature on this subject is controversial. There are studies on cancer patients that describe a significant reduction in physiological parameters, blood pressure and heart rate, after musical interventions^(15-16)^, while others find no differences^(17)^.

Regarding the level of pain, it is true that at the beginning of the musical intervention, patients did not manifest high levels of pain, but in fact reported low levels or no pain at all, which is why no improvement was observed. There is some evidence of this effect in the literature^(18-19)^, while other studies have not identified any differences^(20-21)^. As for the level of anxiety, there was a significant reduction post-intervention. However, it is important to consider that anxiety can decrease considerably after the administration of chemotherapy, so these results should be interpreted with caution.

Furthermore, in our study, the effect size of music on anxiety levels was low. Even so, these findings are in line with other studies^(15,22-23)^, in which live music, compared to other forms of intervention such as pre-recorded music^(17-18,20)^, is associated with a calming effect. One review concluded that musical interventions could reduce anxiety, depression, pain and increase the life expectancy of these patients, improving their physiological and psychological health^(24)^. Although it is true that the evidence for these results is limited, more studies are needed^(25)^.

In the music students, the significant decrease in systolic blood pressure after the rehearsal could reflect a reduction in stress or anticipatory anxiety before the performance. However, during the intervention in the hospital, no significant differences were found in anxiety levels, suggesting that the rehearsal could have a greater impact on reducing anxiety than the intervention itself. Despite the minimal variation in vital signs, it is interesting to note that the physiological parameters decreased when confronted with the hospital environment compared to the previous trials, with the exception of heart rate. The latter could be related to the high level of emotion involved in the experience, which contrasts with the initial hypothesis and reflects the controversy in the literature about this effect.

Regarding the signs and symptoms of anxiety, the performers experienced a higher degree of anxiety before the rehearsal, which decreased at the end of the musical performance. Stage anxiety is a common problem among musicians, which can manifest itself both during their studies and in their professional careers. This anxiety can appear from an early age and have a negative impact on professional and personal performance^(26-27)^. However, there are few studies that include young music students as a population^(26)^.

It is important to include performance strategies for the prevention and self-management of public speaking anxiety during music studies, although there is still little training for this purpose^(27-28)^. However, more recent studies are showing that strategies are being implemented from these early stages to help control stage anxiety in students, the most common of which are simulated interpretation, positive attitude, preparation and breathing, and their teachers play a fundamental role in their professional and emotional training^(26)^.

In this study, the initiative of this type of activity to involve the student body in complex real-life situations stands out, allowing them to develop tools to adapt to and cope with such situations as part of their training. In addition, the aim is to promote student values related to their commitment and responsibility towards health and well-being issues, through teamwork with other disciplines during their academic training. At the same time, the use of artistic modalities, in this case music, improves skills related to empathy, promoting reflection, understanding^(29)^ and social involvement in students^(10,30)^. Therefore, focusing on the arts as an educational tool can be a starting point for developing teaching methodologies that encourage active learning.

In addition, this study encouraged the participation of nursing students in the fieldwork, introducing them to the basic steps of the research. Nurses are in a unique position to identify the care needs of patients and their families, and to plan music-related activities. Thus, early training in this type of intervention, in collaboration with other disciplines that influence well-being, such as music^(10,31)^, is essential to foster holistic care for patients and their environment^(32)^. This model is fundamental in promoting person-centered care, facilitating relationships and support between professionals, patients and family members/caregivers, and favoring the biopsychosocial and spiritual well-being of cancer patients^(33-36)^.

As a limitation, as this is a pilot study, the number of participants is small. Given that the effect of the musical intervention on this specific population was unknown, a small sample size was chosen in order to preliminarily explore the possible effects and feasibility of the study. This approach allows trends to be identified and the study design to be adjusted for future larger studies. It is necessary to continue this project in future academic courses in order to obtain a larger sample and be able to establish relationships between the variables studied and the effect of live music performance. In addition, the musical intervention in this study was limited in time due to the academic calendar and the low participation rate of the patients due to their availability and willingness to take part in the study. Therefore, an increase in the number of sessions, the inclusion of a control group and even a longitudinal follow-up of the study population would enable more robust and conclusive results to be obtained.

Despite the limitations described, music can be an effective tool for reducing anxiety, especially in hospital settings. For students, music is not only an academic discipline, but also a source of emotional well-being. In this sense, it is important to highlight the innovative and interdisciplinary value of this work, which promotes collaboration between students from different degrees, nursing and music, under the coordination of their academic leaders, with the aim of bringing them closer to reality, while giving them the opportunity to enrich their academic curricula. This approach fosters teamwork in an active and collaborative learning environment, encouraging the acquisition and development of communicative, investigative and creative skills from their university studies onwards^(31)^, and these collaborations can be consolidated between them in the future.

It would be beneficial for future research to explore in depth the type of music and the specific conditions that optimize the benefits observed, as well as to investigate the long-term impact of musical interventions. In addition, future studies could focus on the implementation of similar programs in other centers and institutions, assessing their long-term impact on graduates’ professional practice. As far as practical applications are concerned, integrating music as part of complementary therapies in hospital and educational settings could improve the general well-being of the people involved, promoting a more holistic approach to care and education.

## Conclusion

Live music interventions can reduce the signs and symptoms of anxiety and vital signs of both musical performers and cancer patients. In cancer patients, the anxiety sign and symptoms, blood pressure and heart rate decreased after musical intervention. In music students, anxiety signs and symptoms increased, compared to hospital intervention. However, future studies along these lines are needed to confirm these effects.

## Data Availability

The dataset that supports the findings of this study is not publicly available.
