# Supplementary material for: Anxiety and vital signs in cancer patients and music students before and after a music intervention: a quasi-experimental pilot study
Source: Rev Lat Am Enfermagem. 2025 Nov 3;33:e4635. doi: 10.1590/1518-8345.7834.4635 (PMC12587759; doi:10.1590/1518-8345.7834.4635)
Supplement: Translation

# Translation: Ansiedade e sinais vitais de pacientes oncológicos e estudantes de música antes e depois de uma intervencão musical: estudo-piloto quase-experimental*

**DOI:** 10.1590/1518-8345.7834.4636

**Authors:** Raquel Caballero-de-la-Calle, Patricia Ferrero-Sereno, Miguel Ángel García-Garrido, Ana Ruiz-Rodríguez, María Nieves Moro-Tejedor, Miriam Leñero-Cirujano

**Affiliations:** 1 Universidad Alfonso X el Sabio, Facultad de Ciencias Biomédicas y de la Salud, Grado de Enfermería, Madrid, Espanha.; 2 Universidad Alfonso X el Sabio, Facultad de Ciencias Escénicas, Grado de Interpretación Musical, Madrid, Espanha.; 3 Hospital General Universitario Gregorio Marañón, Madrid, Espanha.; 4 Instituto de Investigación Sanitaria Gregorio Marañón, Madrid, Espanha.; 5 Universidad Complutense de Madrid, Facultad de Enfermería, Fisioterapia y Podología, Departamento de Enfermería, Madrid, Espanha.

**Keywords:** Oncologia Integrativa, Enfermagem Holística, Práticas Interdisciplinares, Música, Ansiedade, Sinais Vitais

## Abstract

analisar os sinais e sintomas da ansiedade e os parâmetros dos sinais vitais de pacientes oncológicos e estudantes de música antes e depois de uma intervenção musical ao vivo.

estudo-piloto quase-experimental, sem grupo controle. Participaram 18 pacientes e 10 estudantes de música. A intervenção musical consistiu na interpretação ao vivo, de peças musicais, previamente selecionadas pelos estudantes de música e seus professores responsáveis. Foram avaliados os sinais e sintomas da ansiedade por meio da *Hamilton Anxiety Scale* e dos parâmetros vitais antes e depois da intervenção musical em um hospital-dia. Para os estudantes de música também foram analisadas as variáveis antes e depois do ensaio geral das peças musicais. Foi utilizado o teste de Wilcoxon para a análise dos dados.

a intervenção musical contribuiu para diminuir os sinais e sintomas de ansiedade e os parâmetros vitais dos estudantes e pacientes oncológicos. Para os estudantes de música, os sinais e sintomas de ansiedade foi maior no início do ensaio geral em comparação com a intervenção realizada no hospital.

as intervenções de música ao vivo podem contribuir para melhorar os sinais e sintomas de ansiedade tanto nos pacientes oncológicos quanto para os estudantes de música. Estudos analíticos são necessários para confirmar a relação causa e efeito.

## Destaques

**(1)** A intervenção musical ao vivo incentiva o trabalho colaborativo e a aprendizagem ativa. **(2)** A intervenção musical contribuiu para diminuir a ansiedade e os parâmetros vitais dos estudantes e pacientes oncológicos. **(3)** Os sinais e sintomas de ansiedade foram maiores no ensaio geral em comparação com a intervenção para os estudantes de música. **(4)** Estudos analíticos são necessários para confirmar a relação causa e efeito.

## Introdução

O diagnóstico e tratamento do câncer provocam uma repercussão negativa no bem-estar e na qualidade de vida de quem o sofre. Os pacientes oncológicos, frequentemente, manifestam sentimentos de ansiedade, raiva, medo e tristeza, que se veem exacerbados pela sintomatologia clínica própria da doença, tais como náuseas, vômitos, anorexia, fadiga, insônia, dor, entre outros. Essa combinação de fatores pode gerar neles uma contínua sensação de mal-estar^(1)^.

Nesse sentido, as intervenções voltadas para melhorar o bem-estar e o conforto nesses pacientes são fundamentais^(2-3)^. Um relatório publicado pela Organização Mundial da Saúde (OMS) evidenciou inúmeros estudos que destacavam a importância das artes na saúde^(4)^. Nesse contexto, propõe-se a interpretação de música ao vivo como um recurso holístico e complementar ao tratamento oncológico convencional, com a finalidade de reduzir parte da sintomatologia associada ao processo, aumentar a qualidade de vida desses pacientes e melhorar a relação terapêutica^(5-9)^. Além disso, a musicoterapia está reconhecida como uma intervenção de enfermagem na Classificação de Intervenções de Enfermagem (NIC), definindo-se como a “utilização da música para ajudar a conseguir uma mudança específica, fisiológica, de comportamentos e/ou sentimentos”^(10)^. Essas intervenções musicais não apenas produzem uma repercussão positiva em pacientes, cuidadores e profissionais de saúde, mas também afetam os interpretes, criando uma sinergia enriquecedora entre a música e os cuidados. A interpretação musical ao vivo em ambientes hospitalares representa um desafio para estes últimos, que devem atuar com uma sensibilidade e empatia especiais para se adaptar a um público diferente daquele ao qual normalmente se dirigem em salas de concerto tradicionais^(11)^. São escassos os estudos que consideram essa abordagem triangular pacientes-profissionais de saúde-intérpretes na avaliação do efeito da música nos ambientes de saúde^(11)^.

Neste estudo, essas três vertentes, pacientes-profissionais de saúde-estudantes de música ganham protagonismo, desenvolvendo-se no âmbito de um desafio colaborativo e interdisciplinar entre duas graduações da Universidade Alfonso X El Sabio: Graduação em Enfermagem e Graduações em Interpretação Musical. O objetivo da pesquisa foi analisar os sinais e sintomas da ansiedade e os parâmetros dos sinais vitais de pacientes oncológicos e estudantes antes e depois de uma intervenção musical ao vivo.

## Método

### Tipo de estudo

Estudo-piloto quase-experimental sem grupo controle e sem randomização no qual se propôs analisar os sinais e sintomas de ansiedade e os parâmetros dos sinais vitais de pacientes oncológicos e estudantes de música antes e depois de uma intervenção musical ao vivo.

Nos estudantes de música, também serão avaliadas essas variáveis antes e depois do ensaio geral. Foram seguidas as diretrizes da lista de verificação TIDier (*Template for Intervention Description and Replication*)^(12)^ para o desenvolvimento deste estudo.

Parte-se da hipótese de que a interpretação de música ao vivo em um ambiente hospitalar pode interferir no bem-estar de pacientes e interpretes de intervenções musicais. Nos pacientes oncológicos, espera-se que, após a intervenção musical, haja redução dos sinais e sintomas de ansiedade, e nos estudantes de música, que sejam maiores na intervenção musical ao vivo do que nos ensaios prévios.

### Local e período do estudo

A intervenção foi realizada na unidade do hospital de dia médico oncológico do Hospital Universitário Rey Juan Carlos (HURJC) em Madrid, Espanha. Os ensaios prévios foram realizados nas instalações da Universidade Alfonso X El Sabio (UAX) em Villanueva de la Cañada, Espanha. Esta pesquisa foi desenvolvida durante os meses de fevereiro a maio de 2024, seguindo o calendário acadêmico.

### Participantes

A população de pacientes oncológicos foi composta por aqueles que compareceram ao hospital-dia do HURJC para receber seu ciclo de quimioterapia nos dias selecionados para a interpretação das peças musicais ao vivo. Em média, o hospital recebe aproximadamente 15 pacientes ao dia para tratamento de quimioterapia. O critério de inclusão foi ser adulto com idade igual ou superior a 18 anos. Os critérios de exclusão foram ser pacientes com diagnóstico de demência ou com comprometimento cognitivo de qualquer grau, pacientes com alterações na linguagem de qualquer etiologia que impeçam a comunicação e compreensão e pacientes que já tenham participado de sessões prévias de música ao vivo em nosso estudo.

A população dos intérpretes foi composta pela totalidade de estudantes de música matriculados nos Cursos de Interpretação Musical e Interpretação de Música Moderna (n=10) da UAX, no ano letivo 2023-2024, que concordaram participar voluntariamente do estudo. Foram convidados a participar durante uma breve sessão informativa ao término de uma das aulas habituais.

Tratando-se de um estudo piloto, a amostragem foi oportunista, consecutiva a toda a população acessível, levando em conta os critérios de inclusão e exclusão.

Inicialmente, a amostra foi constituída por 23 pacientes oncológicos, sendo que cinco deles não completaram o estudo devido à finalização antecipada de seu tratamento antes da intervenção musical. Portanto, a amostra foi constituída por 18 pacientes oncológicos e 10 estudantes de música.

### Intervenção da interpretação musical ao vivo

Foi planejada uma atividade educativa de colaboração interdisciplinar entre os estudantes dos Cursos de Interpretação Musical e o Curso de Enfermagem da UAX.

Dois meses antes da intervenção, os estudantes de música realizaram seus ensaios nas instalações da universidade. Uma semana antes da interpretação no hospital, foi realizado o ensaio geral, no qual foram efetuadas a primeira coleta de dados junto aos estudantes de música.

A intervenção musical no hospital consistiu na interpretação ao vivo por solistas ou duos, de peças musicais, previamente selecionadas pelos estudantes de música e seus professores responsáveis, utilizando instrumentos como piano, violino e guitarra. Os critérios para a seleção das peças incluíram a capacidade dessas para promover o relaxamento e o bem-estar emocional dos pacientes: “Scherzo n. º 4 Op. 54” de Chopin para piano solo, a “Habanera” de Bizet e “La vida breve” de Falla para piano a quatro mãos, “Suite in the Old Style” de Schnittke e “Spanish Dance” de Skoryk para duos de violino e piano, e standards de jazz para teclado e guitarra elétrica.

Essas peças eram interpretadas em sessões de 30 minutos, no hospital-dia de oncologia, enquanto os pacientes recebiam seu tratamento quimioterápico. Os dias estabelecidos para a realização das sessões foram mantidos em anonimato para os pacientes, exceto para a direção do hospital e a equipe de enfermagem da unidade, por razões logísticas. Foram realizadas duas intervenções musicais em um dia, repetindo esse mesmo procedimento durante um total de três dias para captar uma maior amostra. A programação da intervenção no hospital foi realizada em conjunto com a direção de enfermagem da instituição. Os pacientes do hospital receberam apenas uma única sessão musical durante o seu ciclo de tratamento.

### Variáveis de estudo e coleta de dados

Foram coletadas variáveis sociodemográficas, tais como, idade, gênero, estado civil e clínicas relacionadas a pressão arterial, frequência cardíaca, saturação de oxigênio e sinais e sintomas de ansiedade para ambos os participantes. A verificação dos sinais vitais foi realizada utilizando tensiômetros e oxímetros de pulso calibrados. Os sinais e sintomas de ansiedade foram avaliados utilizando a escala de ansiedade de *Hamilton Anxiety Scale* (HAS)^(13)^. Essa escala é heteroaplicada e contém 14 itens, dos quais 13 fazem referência a sinais e sintomas ansiosos (estado de ânimo ansioso, tensão, temores, insônia, intelectual/cognitivo, estado de ânimo deprimido, sintomas somáticos gerais [musculares], sintomas somáticos gerais [sensoriais], sintomas cardiovasculares, sintomas respiratórios, sintomas gastrointestinais, sintomas geniturinários e sintomas autônomos) e o último avalia o comportamento do entrevistado, com boa consistência interna (α de Cronbach: 0,88). Cada item tem valores que correspondem de 0 (ausente) a 4 (incapacitante) pontos. Não existem pontos de corte: quanto maior a pontuação na escala, maior a intensidade.

Para os pacientes oncológicos, além da aplicação da HAS foram coletadas outras variáveis clínicas relacionadas à sua patologia, como tipo de tumor, estágio, tratamento atual e nível de dor (avaliado por meio da escala visual analógica). Para os estudantes de música, foi registrada uma avaliação final sobre a experiência (muito boa/boa/regular/ruim/muito ruim) e sua intenção quanto à possível repetição da intervenção (sim/não).

As variáveis sociodemográficas e clínicas foram registradas tanto antes quanto depois da intervenção musical e, no caso dos estudantes, também foram coletadas nos períodos pré e pós-ensaio geral. Para evitar vieses nas respostas, não foi relembrada aos participantes a informação previamente fornecida. Além disso, as identidades dos participantes foram codificadas para garantir o sigilo dos dados.

A coleta dos dados foi realizada por estudantes voluntários do 2º ano do Curso de Enfermagem. Antes da coleta, foi programada uma sessão formativa com os estudantes para informar sobre a natureza do estudo e instruir sobre a adequada coleta das variáveis, seguindo técnicas padronizadas para garantir a precisão e consistência dos dados clínicos.

### Análise dos dados

As variáveis quantitativas foram apresentadas com médias e desvio padrão ou medianas e intervalo interquartílico, conforme sua distribuição. As variáveis qualitativas foram expressas em porcentagens e frequências. Para a comparação das amostras pareadas nos momentos pré e pós intervenção musical, bem como no ensaio, utilizou-se o teste de Wilcoxon, adequado para amostras pequenas que não seguem uma distribuição normal. A análise dos dados foi realizada com o programa estatístico SPSS v.29^(14)^. Considerou-se um valor de p<0,05 para a significância estatística.

### Aspectos éticos

O estudo foi desenvolvido de acordo com a declaração de Helsinki, o cumprimento da Lei de Pesquisa Biomédica e o atendimento às normas de Boa Prática Clínica no que lhe for aplicável, o que inclui o acompanhamento dos participantes do estudo para assegurar a qualidade dos dados e a proteção dos sujeitos participantes. Obteve-se a aprovação por escrito do comitê de ética de ambas as instituições envolvidas no estudo.

Informou-se os pacientes e estudantes sobre o objetivo e o método do trabalho de pesquisa e obteve-se o consentimento livre e esclarecido por escrito. A participação foi de caráter anônimo e voluntário, com o direito do participante de se retirar a qualquer momento. Para preservar a confidencialidade dos dados, foi atribuído um código alfanumérico a cada participante. O tratamento dos dados pessoais foi realizado conforme disposto no Regulamento Geral (UE) 2016/679, de proteção de dados, e na Lei Orgânica 3/2018, de proteção de dados e garantia dos direitos digitais. Este estudo foi financiado na XVI Convocatória de Auxílios para o Desenvolvimento de Projetos de Pesquisa UAX-FUAX 2023.

## Resultados

Dos 18 (100%) pacientes oncológicos, houve paridade entre homens e mulheres, com idades entre 34 e 84 anos, com média de idade de 57,9 (16,3) anos. No caso dos estudantes de música, a amostra foi constituída pela totalidade de alunos matriculados no curso de música, com taxa de participação de 100%. Os estudantes tinham uma média de idade de 22 (1,1) anos, compreendendo entre 19 e 22 anos; desses, 70% eram mulheres (Tabela 1).

**Tabela 1-:** Distribuição numérica e percentual pacientes oncológicos (n* = 18) e estudantes de música (n* = 10) segundo as variáveis sociodemográficas e clínicas. Madrid, Espanha, 2024

Variáveis	Pacientes oncológicos n* (%) ^†^	Estudantes de música n* (%) ^†^
**Gênero**		
Mulher	9 (50%)	7 (70%)
Homem	9 (50%)	3 (30%)
**Estado civil**		
Casado/a	12 (66,7%)	0
Divorciado/a	1 (5,6%)	0
Solteiro/a	4 (22,2%)	10 (100%)
Viúvo/a	1 (5,6%)	0
**Origem do tumor**		
Digestivo	5 (27,8%)	--
Respiratório	7 (38,9%)	--
Ginecológico	5 (27,7%)	--
Sanguíneo	1 (5,6%)	--
**Est** ágio **do tumor**		
I	2 (11,1%)	--
II	0	--
III	9 (50%)	--
IV	7 (38,9%)	--
**Tipo de tratamento quimioterápico**		
Neoadjuvante	8 (44,4%)	--
Adjuvante	2 (11,1%)	--
Paliativo	8 (44,4%)	--

*n = Número de participantes; ^†^Porcentagem

Na Tabela 2 são apresentados os parâmetros vitais na pré e pós-intervenção. Para os pacientes com câncer observa-se um aumento nos valores da pressão arterial sistólica de 12,5 mmHg (p=0,013) e 2,0 mmHg na diastólica (p=0,214), e na frequência cardíaca de 2,5 bpm; (p=0,038) na pós-intervenção em relação à pré-intervenção musical. Não foram observadas alterações no nível de dor. Para os estudantes de música, os valores dos sinais vitais foram muito semelhantes antes e depois do ensaio musical, bem como na intervenção no hospital-dia. Constatou-se diferenças significativas na pressão arterial sistólica antes e depois do ensaio musical, com redução pós-ensaio de 16,5 mmHg em relação ao ensaio musical (p=0,028). Não foram observadas diferenças significativas nos demais parâmetros vitais (p>0,05), bem como entre o pré e o pós-ensaio musical em comparação com o pré e pós-intervenção musical (p>0,05).

**Tabela 2-:** Distribuição das médias dos sinais vitais em pacientes (n* = 18) e estudantes de música (n* = 10) no pré e pós-ensaio musical (estudantes) e no pré e pós-intervenção musical no hospital. Madrid, Espanha, 2024

	Pacientes oncológicos			Estudantes de música					
	Intervenção hospital			Ensaio musical			Intervenção hospital		
Variáveis	Pré	Pós	p valor ^§^	Pré	Pós	p valor ^§^	Pré	Pós	p valor ^§^
	Md ^†^ [RIQ] ^‡^	Md ^†^ [RIQ] ^‡^		Md ^†^ [RIQ] ^‡^	Md ^†^ [RIQ] ^‡^		Md ^†^ [RIQ] ^‡^	Md ^†^ [RIQ] ^‡^	
Pressão arterial sistólica	116,0	128,5	**0,013**	126,5	110,0	**0,028**	116,0	114,5	0,260
	[101,0-133,5]	[107,3-137,3]		[117,5-130,0]	[102,8-122,5]		[111,0-127,0]	[101,8-119,8]	
Pressão arterial diastólica	71,5	73,5	0,214	75,0	73,0	0,779	76,0	73,5	0,359
	[59,5-78,5]	[64,0-82,3]		[65,0-82,5]	[70,0-77,0]		[67,3-86,3]	[66,5-79,5]	
Frequência cardíaca	73,5	76,0	**0,038**	82,5	89,0	0,306	87,00	93,5	0,386
	[66,8-76,0]	[65,8-80,3]		[72,8-93,0]	[68,8-101,0]		[79,0-98,8]	[80,5-102,0]	
Saturação O _2_	97,0	97,0	0,591	98,0	98,0	0,491	97,0	97,0	0,334
	[95,0-98,3]	[94,5-99,3]		[96,8-99,0]	[95,8-98,3]		[94,8-99,3]	[94,8-98,3]	
Nível de dor	1,0	1,0	0,180	--	--	--	--	--	--
	[1,0-2,3]	[1,0-2,3]							

*n = Número de participantes; ^†^Md = Mediana; ^‡^[RIQ] = Intervalo interquartílico; ^§^p-valor do teste de Wilcoxon

Na Tabela 3 é apresentada a distribuição das medianas dos escores dos sinais e sintomas de ansiedade. Para os pacientes oncológicos, em geral, os escores dos sintomas foram menores no pós-intervenção em relação ao pré-intervenção, não se observando diferenças significativas, exceto no item temor (p=0,034) e nos sintomas somáticos gerais (musculares) (p=0,013). No escore total da escala, evidenciou-se redução de 4 pontos pós-intervenção (p=0,004).

**Tabela 3-:** Distribuição das medianas das pontuações de ansiedade em pacientes oncológicos (n* = 18) e estudantes de música (n* = 10) no pré e pós-ensaio musical (estudantes) e no pré e pós-intervenção musical no hospital. Madrid, Espanha, 2024

	Pacientes oncológicos			Estudantes de música					
	Intervenção hospital			Ensaio musical			Intervenção hospital		
Sintomas de ansiedade HAS	Pré	Pós	p valor ^§^	Pré	Pós	p valor ^§^	Pré	Pós	p valor ^§^
	Md ^†^ [RIQ] ^‡^	Md ^†^ [RIQ] ^‡^		Md ^†^ [RIQ] ^‡^	Md ^†^ [RIQ] ^‡^		Md ^†^ [RIQ] ^‡^	Md ^†^ [RIQ] ^‡^	
Estado de ânimo ansioso	1,0	0,0	0,083	2,0	0,0	**0,038**	0,0	0,0	0,317
	[0,0-2,0]	[0,0-1,0]		[0,0-2,0]	[0,0-1,3]		[0,0-1,0]	[0,0-0,3]	
Tensão	0,5	0,0	0,477	0,0	0,0	0,414	0,0	0,0	1
	[0,0-1,3]	[0,0-1,0]		[0,0-2,0]	[0,0-1,3]		[0,0-1,0]	[0,0-1,0]	
Temor	0,5	0,0	**0,034**	1,0	0,0	0,058	0,0	0,0	0,317
	[0,0-1,0]	[0,0-1,0]		[0,8-1,3]	[0,0-0,5]		[0,0-0,0]	[0,0-0,0]	
Insônia	1,0	1,0	0,102	1,0	0,0	**0,024**	0,0	0,0	1
	[0,0-2,0]	[0,0-1,3]		[0,0-2,0]	[0,0-0,3]		[0,0-0,3]	[0,0-0,3]	
Intelectual (cognitivo)	0,0	0,0	0,305	0,0	0,0	0,564	0,0	0,0	1
	[0,0-2,0]	[0,0-1,0]		[0,0-2,3]	[0,0-2,0]		[0,0-0,3]	[0,0-0,3]	
Estado de ânimo deprimido	0,0	0,0	0,058	1,0	0,0	**0,038**	0,0	0,0	1
	[0,0-1,3]	[0,0-1,0]		[0,0-2,0]	[0,0-0,0]		[0,0-0,0]	[0,0-0,0]	
Sintomas somáticos musculares	1,0	0,0	**0,013**	1,5	1,0	0,119	0,0	0,0	0,317
	[0,0-2,0]	[0,0-1,0]		[0,0-2,3]	[0,0-1,3]		[0,0-1,0]	[0,0-0,3]	
Sintomas somáticos sensoriais	1,0	0,0	0,059	0,5	0,0	0,102	0,0	0,0	0,317
	[0,0-1,0]	[0,0-1,0]		[0,0-1,5]	[0,0-1,3]		[0,0-0,0]	[0,0-0,3]	
Sintomas cardiovasculares	0,0	0,0	0,248	0,5	0,0	0,157	0,0	0,0	0,083
	[0,0-1,0]	[0,0-0,3]		[0,0-2,0]	[0,0-2,0]		[0,0-0,3]	[0,0-1,0]	
Sintomas respiratórios	0,0	0,0	0,238	0,0	0,0	1	0,0	0,0	0,317
	[0,0-1,0]	[0,0-0,3]		[0,0-0,5]	[0,0-1,0]		[0,0-0,3]	[0,0-0,0]	
Sintomas gastrointestinais	1,0	0,0	0,405	0,0	0,0	0,157	0,0	0,0	1
	[0,0-1,3]	[0,0-2,0]		[0,0-1,3]	[0,0-1,0]		[0,0-0,3]	[0,0-0,3]	
Sintomas geniturinários	0,0	0,0	0,058	0,0	0,0	1	0,0	0,0	1
	[0,0-1,0]	[0,0-0,0]		[0,0-0,0]	[0,0-0,0]		[0,0-0,0]	[0,0-0,0]	
Sintomas autônomos	1,0 [0,0-1,3]	0,0	0,058	1,0	0,0	0,655	0,0	0,5	0,564
		[0,0-1,0]		[0,0-1,0]	[0,0-2,0]		[0,0-1,3]	[0,0-1,0]	
Comportamento na entrevista	1,0	0,5	0,739	1,5	1,0	0,488	0,50	1,0	0,059
	[0,0-1,0]	[0,0-1,0]		[0,0-2,0]	[0,0-2,0]		[0,00-1,00]	[0,0-2,0]	
HAS total	9,5	5,5	**0,004**	11,5	5,0	**0,019**	2,5	4,0	0,269
	[8,0-15,3]	[3,0-9,3]		[5,8-18,8]	[0,7-14,0]		[0,0-7,8]	[0,0-7,3]	

*n = Número de participantes; ^†^Md = Mediana; ^‡^[RIQ] = Intervalo interquartílico; ^§^p-valor teste de Wilcoxon

Para os estudantes de música, no ensaio geral, observou-se uma redução na maioria dos sinais e sintomas de ansiedade ao final da interpretação em comparação com o início do ensaio musical, sendo as diferenças significativas nos sintomas: estado de ânimo ansioso (p=0,038), insônia (p=0,024) e estado de ânimo deprimido (p=0,038). Contudo, na intervenção musical realizada no hospital-dia, os escores dos sinais e sintomas de ansiedade foram muito semelhantes entre si, não se observando diferenças estatisticamente significativas (p>0,005).

Tampouco foram encontradas diferenças entre o ensaio musical e q intervenção no hospital (p>0,005), mas sim entre os dois momentos anteriores em ambos os ambientes, especificamente nos itens: estado de ânimo ansioso (p=0,024), temor (p=0,008), estado de ânimo deprimido (p=0,038), sintomas somáticos musculares (p=0,031), sintomas somáticos sensoriais (p=0,038), comportamento na entrevista (p=0,034) e na pontuação total da HAS (p=0,012). Nesta última, evidenciou-se que os sinais e sintomas de ansiedade eram significativamente maiores no início do ensaio geral (11,5; p=0,019) em comparação com os demais momentos de medição, tanto após o ensaio (5,0; p=0,019) quanto nos momentos antes e depois da intervenção musical (2,50 e 4,0, respectivamente; p=0,012). Os sinais e sintomas de ansiedade foram ligeiramente maiores ao final da intervenção do que em seu início, embora essa diferença não tenha sido estatisticamente significativa (p=0,269) (Tabela 3).

Quanto à avaliação da experiência, todos os estudantes de música a avaliaram como muito boa (80%) e boa (20%), sendo que a totalidade dos estudantes estariam dispostos a participar novamente, caso a intervenção se repetisse em cursos posteriores.

## Discussão

Neste estudo são apresentados os resultados da implementação de uma intervenção educacional de inovação interdisciplinar, que integra as artes e as ciências da saúde, no contexto do cuidado biopsicossocial e do trabalho em equipe dos estudantes em um ambiente sanitário real. Os resultados oferecem uma visão interessante sobre o papel da música no bem-estar físico, psicológico e emocional, destacando seu potencial como ferramenta complementar na área da saúde.

O aumento significativo da pressão arterial sistólica e da frequência cardíaca nos pacientes oncológicos após a intervenção, em comparação à pré-intervenção, poderia dever-se a uma resposta fisiológica ao estímulo musical. No entanto, essa variação nas constantes vitais não pode ser atribuída exclusivamente à intervenção, já que pode estar relacionada pela administração do tratamento quimioterápico, entre outros fatores. A literatura sobre este tema mostra-se controversa. Existem estudos em pacientes oncológicos que descrevem uma redução significativa nos parâmetros fisiológicos, pressão arterial e frequência cardíaca, após intervenções musicais^(15-16)^, enquanto outros não encontram diferenças^(17)^.

Em relação ao nível de dor, é verdade que, no início da intervenção musical, os pacientes não manifestaram níveis elevados de dor, tendo, na verdade, relatado níveis baixos ou ausência de dor, razão pela qual não se observaram melhoras. Na literatura, encontram-se algumas evidências sobre esse efeito^(18-19)^, enquanto outros estudos não identificaram diferenças^(20-21)^. Quanto à presença de sinais e sintomas de ansiedade, observou-se uma diminuição significativa na pós-intervenção. No entanto, é importante considerar que a ansiedade pode diminuir consideravelmente após a administração da quimioterapia, pelo que esses resultados devem ser interpretados com cautela.

Ademais, em nosso estudo, o tamanho do efeito da música nos sinais e sintomas de ansiedade foi baixo. Ainda assim, esses achados estão em consonância com outras pesquisas^(15,22-23)^, nas quais a música ao vivo, em comparação com outras formas de intervenção, como a música pré-gravada^(17-18,20)^, está associada a um efeito calmante. Em uma revisão, concluiu-se que as intervenções musicais poderiam diminuir a ansiedade, a depressão, a dor e aumentar a expectativa de vida desses pacientes, melhorando sua saúde fisiológica e psicológica^(24)^. Embora seja verdade que a evidência desses resultados é limitada, são necessários mais estudos^(25)^.

Nos estudantes de música, a diminuição significativa na pressão arterial sistólica após o ensaio poderia refletir uma redução do estresse ou da ansiedade antecipatória antes da interpretação. Contudo, durante a intervenção no hospital, não se encontraram diferenças significativas quanto aos sinais e sintomas de ansiedade, o que sugere que o ensaio poderia ter um impacto maior na redução desta do que a própria intervenção. Apesar da variação mínima nos sinais vitais, é interessante destacar que os parâmetros fisiológicos diminuíram ao confrontar o ambiente hospitalar em comparação com os ensaios prévios, exceto a frequência cardíaca. Esta última poderia estar relacionada ao alto grau emocional que a experiência implicava, o que contrasta com a hipótese inicial e reflete a controvérsia existente na literatura sobre esse efeito.

Em relação à presença de sinais e sintomas de ansiedade, os intérpretes experimentaram maior intensidade de ansiedade antes do ensaio, o qual diminuiu ao término da interpretação musical. A ansiedade cênica é um problema comum entre os músicos, que pode manifestar-se tanto durante seus estudos quanto em sua carreira profissional. Essa ansiedade pode surgir desde idades precoces e ter um impacto negativo no desempenho profissional e pessoal^(26-27)^. No entanto, são escassos os estudos que incluem como população estudantes de música jovens^(26)^.

Ressalta-se a importância de incluir, durante os estudos musicais, estratégias de atuação para a prevenção e autogestão da ansiedade em público, sendo ainda hoje escassas as formações com esse objetivo^(27-28)^. No entanto, os estudos mais recentes estão evidenciando a implementação de estratégias desde essas etapas precoces para ajudar a controlar a ansiedade cênica nos estudantes, sendo as mais comuns a interpretação simulada, a atitude positiva, a preparação e a respiração, e seus professores desempenham um papel fundamental em sua formação profissional e emocional^(26)^.

Neste estudo, destaca-se a iniciativa desse tipo de atividade de participação do corpo discente em situações reais complexos, que lhes permite desenvolver ferramentas para se adaptar e enfrentar tais situações como parte de sua formação. Além disso, pretende-se promover valores no estudante relacionados ao seu compromisso e responsabilidade com temas sobre saúde e bem-estar, por meio do trabalho em equipe com outras disciplinas durante sua formação acadêmica. Paralelamente, o uso de modalidades artísticas, neste caso a música, melhora as habilidades relacionadas à empatia, promovendo nos estudantes a reflexão, a compreensão^(29)^ e sua implicação social^(10,30)^. Por isso, o enfoque nas artes como ferramenta educacional pode ser um ponto de partida para desenvolver metodologias docentes que incentivem a aprendizagem ativa.

Ademais, neste estudo foi incentivada a participação de estudantes de enfermagem no trabalho de campo, introduzindo-os aos passos elementares da pesquisa. As enfermeiras estão em posição única para identificar as necessidades de atenção dos pacientes e de suas famílias, e para planejar atividades relacionadas à música. Dessa forma, a formação precoce, nesse tipo de intervenção, em colaboração com outras disciplinas influentes no bem-estar, como a música^(10,31)^, é essencial para fomentar o cuidado holístico do paciente e de seu entorno^(32)^. Esse modelo é fundamental na promoção da atenção centrada na pessoa, facilitando as relações e o apoio entre profissionais, pacientes e familiares/cuidadores, e favorecendo o bem-estar biopsicossocial e espiritual dos pacientes oncológicos^(33-36)^.

Como limitações, por se tratar de um estudo-piloto, o número de participantes é reduzido. Dado que se desconhecia o efeito da intervenção musical nessa população específica, optou-se por um tamanho amostral reduzido para explorar preliminarmente os possíveis efeitos e a viabilidade do estudo. Esse enfoque permite identificar tendências e ajustar o desenho do estudo para futuras pesquisas mais amplas. É necessário dar continuidade a esse projeto em futuros cursos acadêmicos para se obter uma amostra maior e poder estabelecer relações entre as variáveis estudadas e o efeito da interpretação de música ao vivo. Além disso, a intervenção musical neste estudo está condicionada à sua limitação temporal, por motivos do calendário acadêmico e pela baixa taxa de participação dos pacientes, devido à sua disponibilidade e disposição para participar do estudo. Portanto, um aumento no número de sessões, a inclusão de um grupo controle e até mesmo um acompanhamento longitudinal da população de estudo permitiriam obter resultados mais robustos e conclusivos.

Apesar das limitações descritas, a música pode ser uma ferramenta eficaz para reduzir a ansiedade, especialmente em contextos hospitalares. Para os estudantes, a música não é apenas uma disciplina acadêmica, mas também uma fonte de bem-estar emocional. Nesse sentido, é importante destacar o valor inovador e interdisciplinar deste trabalho, que promove a colaboração entre estudantes de diferentes graduações, enfermagem e música, sob a coordenação de seus responsáveis acadêmicos, com o objetivo de aproximá-los da realidade, ao mesmo tempo em que lhes é concedida a oportunidade de enriquecer seus currículos acadêmicos. Esse enfoque fomenta o trabalho em equipe em um ambiente de aprendizagem ativa e colaborativa, impulsionando a aquisição e o desenvolvimento de habilidades comunicativas, investigativas e criativas desde seus estudos universitários^(31)^, podendo essas colaborações se consolidarem entre eles no futuro.

Em pesquisa futura seria importante explorar em profundidade o tipo de música e as condições específicas que otimizam os benefícios observados, bem como investigar o impacto a longo prazo das intervenções musicais. Além disso, estudos futuros poderiam se concentrar na implementação de programas similares em outros centros e instituições, avaliando seu impacto a longo prazo na prática profissional dos graduados. No que diz respeito às aplicações práticas, integrar a música como parte das terapias complementares em ambientes hospitalares e educacionais poderia melhorar o bem-estar geral das pessoas envolvidas, promovendo um enfoque mais holístico do cuidado e da educação.

## Conclusão

As intervenções de música ao vivo podem contribuir para redução dos sinais e sintomas de ansiedade e dos parâmetros vitais dos alunos e dos pacientes oncológicos. Nos pacientes oncológicos, os sinais e sintomas de ansiedade, bem como a pressão arterial e os batimentos cardíacos diminuíram após a intervenção musical. Nos estudantes de música, os sinais e sintomas de ansiedade aumentaram no ensaio geral em comparação com a intervenção no hospital. No entanto, estudos analíticos são necessários para confirmar a relação causa e efeito.

## Data Availability

O conjunto de dados que dá suporte aos resultados deste estudo não está disponível publicamente.

# Translation: Ansiedad y signos vitales de pacientes con cáncer y estudiantes de música antes y después de una intervención musical: un estudio piloto cuasiexperimental*

**DOI:** 10.1590/1518-8345.7834.4634

**Authors:** Raquel Caballero-de-la-Calle, Patricia Ferrero-Sereno, Miguel Ángel García-Garrido, Ana Ruiz-Rodríguez, María Nieves Moro-Tejedor, Miriam Leñero-Cirujano

**Affiliations:** 1 Universidad Alfonso X el Sabio, Facultad de Ciencias Biomédicas y de la Salud, Grado de Enfermería, Madrid, España.; 2 Universidad Alfonso X el Sabio, Facultad de Ciencias Escénicas, Grado de Interpretación Musical, Madrid, España.; 3 Hospital General Universitario Gregorio Marañón, Madrid, España.; 4 Instituto de Investigación Sanitaria Gregorio Marañón, Madrid, España.; 5 Universidad Complutense de Madrid, Facultad de Enfermería, Fisioterapia y Podología, Departamento de Enfermería, Madrid, España.

**Keywords:** Oncología Integrativa, Enfermería Holística, Prácticas Interdisciplinarias, Música, Ansiedad, Signos Vitales

## Abstract

analizar los signos y síntomas de ansiedad y los parámetros de signos vitales en pacientes con cáncer y estudiantes de música antes y después de una intervención musical en vivo.

estudio piloto cuasi-experimental, sin grupo control. Participaron 18 pacientes con cáncer y 10 estudiantes de música. La intervención musical consistió en la interpretación en vivo de piezas musicales previamente seleccionadas por los alumnos de música y sus profesores responsables. Se evaluaron los signos y síntomas de ansiedad utilizando la Escala de Ansiedad de Hamilton y parámetros vitales antes y después de la intervención musical en un hospital de día. Para los estudiantes de música también se analizaron variables antes y después del ensayo general de las piezas musicales. Para analizar los datos se utilizó la prueba de Wilcoxon.

se observaron cambios en los parámetros vitales en pacientes con cáncer y estudiantes de música. La intervención musical contribuyó a reducir los signos y síntomas de ansiedad y parámetros vitales de estudiantes y pacientes con cáncer. En los estudiantes de música, los signos y síntomas de ansiedad fueron mayores al inicio del ensayo general en comparación con la intervención realizada en el hospital.

las intervenciones de música en vivo pueden contribuir a mejorar los signos y síntomas de ansiedad tanto en pacientes con cáncer como en estudiantes de música. Se necesitan estudios analíticos para confirmar la relación de causa y efecto.

## Destacados

**(1)** La intervención musical en vivo fomenta el trabajo colaborativo y el aprendizaje activo. **(2)** La intervención musical contribuyó a reducir la ansiedad y los parámetros vitales de estudiantes y pacientes con cáncer. **(3)** Los signos y síntomas de ansiedad fueron mayores en el ensayo general en comparación con la intervención para estudiantes de música. **(4)** Se necesitan estudios analíticos para confirmar la relación causa-efecto.

## Introducción

El diagnóstico y tratamiento del cáncer tiene repercusión negativa el bienestar y la calidad de vida de las personas afectadas. Los pacientes oncológicos a menudo manifiestan sentimientos de ansiedad, ira, temor y tristeza, que se agravan debido a la sintomatología clínica propia de la enfermedad, como náuseas, vómitos, anorexia, fatiga, insomnio, dolor, entre otros. Esta combinación de factores puede ocasionar en ellos una persistente sensación de malestar^(1)^.

En este sentido, las intervenciones dirigidas a mejorar el bienestar y el confort de los pacientes oncológicos resultan fundamentales^(2-3)^. Un informe publicado por la Organización Mundial de la Salud (OMS) destacó numerosos estudios que evidencian la importancia de las artes en la salud^(4)^. En este contexto, se propone la interpretación de música en directo como un recurso holístico y complementario al tratamiento convencional oncológico, capaz de reducir parte de la sintomatología asociada, aumentar la calidad de vida de los pacientes y fortalecer la relación terapéutica^(5-9)^. Además, la musicoterapia está reconocida como una intervención enfermera en la Clasificación de Intervenciones de Enfermería (NIC), definiéndose como la “utilización de la música para ayudar a conseguir un cambio específico, fisiológico, de conductas y/o sentimientos”^(10)^. Estas intervenciones musicales no solo generan un impacto positivo en pacientes, cuidadores y profesionales sanitarios, sino que también benefician a los intérpretes, creando una sinergia enriquecedora entre la música y los cuidados. Cabe destacar que la interpretación musical en vivo en entornos hospitalarios supone un desafío para los intérpretes, quiénes deben actuar con una sensibilidad y empatía especiales para adaptarse a una audiencia diferente a la de salas de conciertos tradicionales^(11)^. Son escasos los estudios que han abordado este enfoque triangular –pacientes, profesionales sanitarios e estudiantes de música– en la evaluación del efecto de la música en los entornos sanitarios^(11)^.

En este estudio, toman protagonismo tres vertientes –pacientes, profesionales sanitarios e estudiantes de música–, desarrollándose en el marco de un reto colaborativo e interdisciplinar entre dos titulaciones de la Universidad Alfonso X el Sabio: el Grado en Enfermería y los Grados en Interpretación Musical. El objetivo principal de la investigación fue analizar los signos y síntomas de ansiedad y los parámetros de signos vitales en pacientes con cáncer y estudiantes de música antes y después de una intervención musical en vivo.

## Método

### Tipo de estudio

Estudio piloto cuasiexperimental sin grupo control y sin aleatorización, en el cual se propuso analizar los signos y síntomas de la ansiedad y los parámetros de signos vitales de pacientes con cáncer y estudiantes de música antes y después de una intervención musical en vivo. En estos últimos, también se evalúan dichas variables antes y después del ensayo general. Se siguieron las directrices de la lista de verificación TIDier (*Template for Intervention Desscription and Replication*)^(12)^ para el desarrollo de este estudio.

La hipótesis es que la interpretación de música en vivo en un entorno hospitalario puede interferir en el bienestar de los pacientes y de los intérpretes de intervenciones musicales. En pacientes con cáncer, se espera que, tras la intervención musical, se produzca una reducción de los signos y síntomas de ansiedad, y en los estudiantes de música, que sean mayores en la intervención musical en vivo que en los ensayos previos.

### Ámbito y periodo de estudio

La intervención se llevó a cabo en la unidad del hospital de día médico oncológico del Hospital Universitario Rey Juan Carlos (HURJC), en Madrid, España. Los ensayos previos se realizaron en las instalaciones de la Universidad Alfonso X el Sabio (UAX), en Villanueva de la Cañada, España. Esta investigación se desarrolló durante los meses de febrero a mayo de 2024, en consonancia con el calendario académico.

### Participantes

La población de pacientes con cáncer estuvo conformada por aquellos que acudieron al hospital de día del HURJC para recibir su ciclo de quimioterapia en los días seleccionados para la interpretación de las piezas musicales *in vivo*. En promedio, el hospital recibe aproximadamente 15 pacientes diarios para tratamiento de quimioterapia. El criterio de inclusión fue ser adulto mayor de 18 años. Los criterios de exclusión fueron: pacientes con diagnóstico de demencia o deterioro cognitivo de cualquier grado; pacientes con alteraciones del lenguaje de cualquier etiología que impidan la comunicación y comprensión; y aquellos que ya hayan participado en sesiones previas de música en directo en nuestro estudio.

La población de los intérpretes estuvo conformada por todos los estudiantes de música matriculados en los Grados Interpretación Musical e Interpretación de Música Moderna (N=10) de la UAX en el curso académico 2023-2024, que accedieron a participar voluntariamente en el estudio. Se les invitó a participar durante una breve sesión informativa al finalizar una de las clases habituales.

Al tratarse de un estudio piloto, el tipo de muestreo fue oportunista, consecutivo a toda la población accesible, teniendo en cuenta los criterios de inclusión y exclusión.

Inicialmente, la muestra estuvo compuesta por 23 pacientes con cáncer, cinco de los cuales no completaron el estudio debido a la finalización prematura de su tratamiento antes de la intervención musical. Por tanto, la muestra estuvo compuesta por 18 pacientes con cáncer y 10 estudiantes de música.

### Intervención de la interpretación musical en directo

Se planificó una actividad educativa de colaboración interdisciplinar entre los estudiantes de los Grados de Interpretación Musical y el Grado de Enfermería de la UAX. Dos meses antes de la intervención, los estudiantes de música realizaron sus ensayos en las instalaciones de la universidad. Una semana antes de la interpretación en el hospital, se llevó a cabo el ensayo general, en el cual se efectuaron la primera recopilación de datos de estudiantes de música.

La intervención musical en el hospital consistió en interpretación en directo, a cargo de solistas o dúos, de piezas previamente seleccionadas por los estudiantes de música y sus profesores, utilizando piano, violín y guitarra. Los criterios de selección priorizaron obras capaces de promover la relajación y el bienestar emocional de los pacientes. La programación incluyó: “Scherzo n.º 4 Op. 54” de Chopin (piano solista);“Habanera” de Bizet y “La vida breve” de Falla (piano a cuatro manos), “Suite in the Old Style” de Schnittke y “Spanish Dance” de Skoryk (dúo de violín y piano); y estándares de jazz para teclado y guitarra eléctrica.

Estas piezas se interpretaron en sesiones de 30 minutos en el hospital de día oncológico, mientras los pacientes recibían su tratamiento quimioterápico. Los días establecidos para las intervenciones se mantuvieron en anonimato para los pacientes, con excepción de la dirección del hospital y el equipo de enfermería, por razones logísticas. Se realizaron dos sesiones diarias, repitiendo el procedimiento durante tres días consecutivos para aumentar el tamaño de la muestra. La planificación de la intervención en el hospital se llevó a cabo en colaboración con la dirección de enfermería de la institución. Cada paciente asistió a una única sesión musical durante su ciclo de tratamiento.

### Variables de estudio y recolección de datos

Se recogieron variables sociodemográficas: edad, género, estado civil; y clínicas: tensión arterial, frecuencia cardiaca, saturación de oxígeno y signos y síntomas de ansiedad para ambos participantes. Las mediciones de los signos vitales se realizaron utilizando tensiómetros y oxímetros de pulso. Los signos y síntomas de ansiedad se evaluaron utilizando la Escala de Ansiedad de Hamilton (HAS)^(13)^. Esta escala es heteroadministrada y contiene 14 ítems, de los cuales 13 se refieren a signos y síntomas de ansiedad – como estado de ánimo ansioso, tensión, temores, insomnio, intelectual/cognitivo, estado de ánimo deprimido, síntomas somáticos generales (musculares), síntomas somáticos generales (sensoriales), síntomas cardiovasculares, síntomas respiratorios, síntomas gastrointestinales, síntomas genitourinarios y síntomas autónomos–; y el ítem 14 valora el comportamiento del entrevistado. Cada ítem tiene valores que corresponden de 0 (ausente) a 4 (incapacitante), sin puntos de corte: a mayor puntuación, mayor intensidad de ansiedad. La escala presenta una buena consistencia interna (α de Cronbach: 0,88).

Para los pacientes con cáncer, se recogieron también variables clínicas específicas de su patología: tipo de tumor, estadio, tratamiento actual y nivel del dolor, este último evaluado mediante la escala visual analógica. Para los estudiantes de música, se registró una valoración final sobre la experiencia (muy buena, buena, regular, mala o muy mala) y su intención de repetir la intervención (sí/no).

Las variables sociodemográficas y clínicas se recopilaron antes y después de la intervención musical; en el caso de los estudiantes, también se midieron en los periodos pre y post ensayo general. Para minimizar sesgos, no se recordó a los participantes los datos ya registrados y se codificaron sus identidades para garantizar el anonimato.

La recopilación de los datos fue realizada por estudiantes voluntarios del segundo curso del Grado en Enfermería. Antes de empezar, se organizó una sesión formativa para explicar la naturaleza del estudio e instruirles en la correcta recogida de variables. Durante la sesión, se revisaron técnicas estandarizadas para asegurar la precisión y consistencia de los datos clínicos.

### Análisis de datos

Las variables cuantitativas se describieron como media ± DE o mediana (RIC) según su distribución, y las variables cualitativas se expresaron como frecuencias absolutas y relativas (%). Para comparar las muestras pareadas, tanto en los momentos pre y post intervención musical como durante el ensayo general, se empleó la prueba de rangos con signo de Wilcoxon, adecuada para muestras pequeñas sin distribución normal. El análisis de los datos se llevó a cabo con el programa estadístico SPSS v.29^(14)^ y se consideró significativo un valor de p<0,05.

### Aspectos éticos

El estudio se desarrolló conforme a la Declaración de Helsinki, a la Ley de Investigación Biomédica y las normas de Buenas Prácticas Clínicas. Estas últimas incluyen el seguimiento de las participantes para garantizar tanto la calidad de los datos como la protección de los participantes. Se obtuvo aprobación por escrito de los comités de ética de ambas instituciones involucradas en el estudio.

Se informó a los pacientes y estudiantes sobre el objetivo y el método del estudio, y se obtuvo su consentimiento informado. La participación fue anónima y voluntaria, con derecho a retirarse en cualquier momento. Para proteger la confidencialidad, se asignó un código alfanumérico a cada participante. El tratamiento de los datos personales se realizó de conformidad con el Reglamento General (UE) 2016/679 de protección de datos y la Ley Orgánica 3/2018 de Protección de Datos y Garantía de los Derechos Digitales. Este estudio recibió financiación de la XVI Convocatoria de Ayudas para el Desarrollo de Proyectos de Investigación UAX -FUAX 2023.

## Resultados

La muestra final quedó constituida por 18 pacientes con cáncer, con distribución paritaria por género y edades comprendidas entre 34 y 84 años (57,9 ± 16,3 años). En el grupo de estudiantes participaron los 10 alumnos matriculados en el Grado de Música de la UAX (tasa de participación del 100%). Su edad media fue de 22 ± 1,1 años [19-22 años] y el 70% fueron mujeres (Tabla 1).

**Tabla 1-:** Distribución numérica y porcentual de pacientes con cáncer (n* = 18) y estudiantes de música (n* = 10) según variables sociodemográficas y clínicas. Madrid, España, 2024

Variables	Pacientes con cáncer n* (%) ^†^	Estudiantes de música n* (%) ^†^
**Género**		
Mujer	9 (50%)	7 (70%)
Hombre	9 (50%)	3 (30%)
**Estado civil**		
Casado/a	12 (66,7%)	0
Divorciado/a	1 (5,6%)	0
Soltero/a	4 (22,2%)	10 (100%)
Viudo/a	1 (5,6%)	0
**Origen tumor**		
Digestivo	5 (27,8%)	--
Respiratorio	7 (38,9%)	--
Ginecológico	5 (27,7%)	--
Sanguíneo	1 (5,6%)	--
**Estadio tumor**		
I	2 (11,1%)	--
II	0	--
III	9 (50%)	--
IV	7 (38,9%)	--
**Tipo tratamiento quimioterápico**		
Neoadyuvante	8 (44,4%)	--
Adyuvante	2 (11,1%)	--
Paliativa	8 (44,4%)	--

*n = Número de participantes; ^†^Porcentaje

En la Tabla 2 se muestran los parámetros vitales antes y después de la intervención. En los pacientes con cáncer se observó un aumento en los valores de presión arterial sistólica de 12,5 mmHg (p=0,013) y 2,0 mmHg en la diastólica (p=0,214) y en la frecuencia cardíaca de 2,5 lpm; (p=0,038) en la postintervención en relación a la preintervención musical. No se observaron cambios en el nivel de dolor. Para los estudiantes de música, el valor de los parámetros vitales fue muy similar antes y después del ensayo musical, así como en la intervención en el hospital. Se observaron diferencias significativas en la presión arterial sistólica antes y después del ensayo, con una reducción post ensayo de 16,5 mmHg en relación al momento previo al mismo (p=0,028). No se observaron diferencias significativas en el resto de parámetros vitales (p>0,05).

**Tabla 2-:** Distribución de los promedios de los signos vitales en los pacientes con cáncer (n* = 18) y estudiantes de música (n* = 10) en el pre-post ensayo musical (estudiantes) y pre-post intervención musical en el hospital de día. Madrid, España, 2024

	**Pacientes con cáncer**			**Estudiantes de música**					
	**Intervención hospital**			**Ensayo musical**			**Intervención hospital**		
**Variables**	**Pre**	**Post**	**p valor** ^§^	**Pre**	**Post**	**p valor** ^§^	**Pre**	**Post**	**p valor** ^§^
	Md ^†^ [RIQ] ^‡^	Md ^†^ [RIQ] ^‡^		Md ^†^ [RIQ] ^‡^	Md ^†^ [RIQ] ^‡^		Md ^†^ [RIQ] ^‡^	Md ^†^ [RIQ] ^‡^	
Presión arterial sistólica	116,0	128,5	**0,013**	126,5	110,0	**0,028**	116,0	114,5	0,260
	[101,0-133,5]	[107,3-137,3]		[117,5-130,0]	[102,8-122,5]		[111,0-127,0]	[101,8-119,8]	
Presión arterial diastólica	71,5	73,5	0,214	75,0	73,0	0,779	76,0	73,5	0,359
	[59,5-78,5]	[64,0-82,3]		[65,0-82,5]	[70,0-77,0]		[67,3-86,3]	[66,5-79,5]	
Frecuencia cardíaca	73,5	76,0	**0,038**	82,5	89,0	0,306	87,00	93,5	0,386
	[66,8-76,0]	[65,8-80,3]		[72,8-93,0]	[68,8-101,0]		[79,0-98,8]	[80,5-102,0]	
Saturación O _2_	97,0	97,0	0,591	98,0	98,0	0,491	97,0	97,0	0,334
	[95,0-98,3]	[94,5-99,3]		[96,8-99,0]	[95,8-98,3]		[94,8-99,3]	[94,8-98,3]	
Nivel de dolor	1,0	1,0	0,180	--	--	--	--	--	--
	[1,0-2,3]	[1,0-2,3]							

*n = Número de participantes; ^†^Md = Mediana; ^‡^[RIQ] = Rango Intercuartílico; ^§^p-valor test de Wilcoxon

Los estadísticos relativos a la distribución de las medianas de las puntuaciones y los signos y síntomas de la escala de ansiedad se presentan en la Tabla 3. En la muestra de pacientes, la puntuación de los ítems era más pequeña en la postintervención que en la preintervención, sin diferencias significativas, salvo en el ítem temor (p=0,034) y en los síntomas somáticos generales (musculares) (p=0,013). Además, en la puntuación total de la escala se evidenció una disminución de 4 puntos postintervención (p=0,004).

**Tabla 3-:** Distribución de las medianas de las puntuaciones de ansiedad en los pacientes con cáncer (n* = 18) y estudiantes de música (n* = 10) en el pre-post ensayo musical (estudiantes) y pre-post intervención musical en el hospital. Madrid, España, 2024

	**Pacientes con cáncer**			**Estudiantes de música**					
	**Intervención hospital**			**Ensayo musical**			**Intervención hospital**		
Ítems escala **HAS**	**Pre**	**Post**	**p valor** ^§^	**Pre**	**Post**	**p valor** ^§^	**Pre**	**Post**	**p valor** ^§^
	Md ^†^ [RIQ] ^‡^	Md ^†^ [RIQ] ^‡^		Md ^†^ [RIQ] ^‡^	Md ^†^ [RIQ] ^‡^		Md ^†^ [RIQ] ^‡^	Md ^†^ [RIQ] ^‡^	
Estado de ánimo ansioso	1,0	0,0	0,083	2,0	0,0	**0,038**	0,0	0,0	0,317
	[0,0-2,0]	[0,0-1,0]		[0,0-2,0]	[0,0-1,3]		[0,0-1,0]	[0,0-0,3]	
Tensión	0,5	0,0	0,477	0,0	0,0	0,414	0,0	0,0	1
	[0,0-1,3]	[0,0-1,0]		[0,0-2,0]	[0,0-1,3]		[0,0-1,0]	[0,0-1,0]	
Temor	0,5	0,0	**0,034**	1,0	0,0	0,058	0,0	0,0	0,317
	[0,0-1,0]	[0,0-1,0]		[0,8-1,3]	[0,0-0,5]		[0,0-0,0]	[0,0-0,0]	
Insomnio	1,0	1,0	0,102	1,0	0,0	**0,024**	0,0	0,0	1
	[0,0-2,0]	[0,0-1,3]		[0,0-2,0]	[0,0-0,3]		[0,0-0,3]	[0,0-0,3]	
Intelectual (cognitivo)	0,0	0,0	0,305	0,0	0,0	0,564	0,0	0,0	1
	[0,0-2,0]	[0,0-1,0]		[0,0-2,3]	[0,0-2,0]		[0,0-0,3]	[0,0-0,3]	
Deprimido	0,0	0,0	0,058	1,0	0,0	**0,038**	0,0	0,0	1
	[0,0-1,3]	[0,0-1,0]		[0,0-2,0]	[0,0-0,0]		[0,0-0,0]	[0,0-0,0]	
Síntomas somáticos musculares	1,0	0,0	**0,013**	1,5	1,0	0,119	0,0	0,0	0,317
	[0,0-2,0]	[0,0-1,0]		[0,0-2,3]	[0,0-1,3]		[0,0-1,0]	[0,0-0,3]	
Síntomas somáticos sensoriales	1,0	0,0	0,059	0,5	0,0	0,102	0,0	0,0	0,317
	[0,0-1,0]	[0,0-1,0]		[0,0-1,5]	[0,0-1,3]		[0,0-0,0]	[0,0-0,3]	
Síntomas cardio-vasculares	0,0	0,0	0,248	0,5	0,0	0,157	0,0	0,0	0,083
	[0,0-1,0]	[0,0-0,3]		[0,0-2,0]	[0,0-2,0]		[0,0-0,3]	[0,0-1,0]	
Síntomas respiratorios	0,0	0,0	0,238	0,0	0,0	1	0,0	0,0	0,317
	[0,0-1,0]	[0,0-0,3]		[0,0-0,5]	[0,0-1,0]		[0,0-0,3]	[0,0-0,0]	
Síntomas gastrointestinales	1,0	0,0	0,405	0,0	0,0	0,157	0,0	0,0	1
	[0,0-1,3]	[0,0-2,0]		[0,0-1,3]	[0,0-1,0]		[0,0-0,3]	[0,0-0,3]	
Síntomas genitourinarios	0,0	0,0	0,058	0,0	0,0	1	0,0	0,0	1
	[0,0-1,0]	[0,0-0,0]		[0,0-0,0]	[0,0-0,0]		[0,0-0,0]	[0,0-0,0]	
Síntomas autónomos	1,0 [0,0-1,3]	0,0	0,058	1,0	0,0	0,655	0,0	0,5	0,564
		[0,0-1,0]		[0,0-1,0]	[0,0-2,0]		[0,0-1,3]	[0,0-1,0]	
Comportamiento en la entrevista	1,0	0,5	0,739	1,5	1,0	0,488	0,50	1,0	0,059
	[0,0-1,0]	[0,0-1,0]		[0,0-2,0]	[0,0-2,0]		[0,00-1,00]	[0,0-2,0]	
HAS total	9,5	5,5	**0,004**	11,5	5,0	**0,019**	2,5	4,0	0,269
	[8,0-15,3]	[3,0-9,3]		[5,8-18,8]	[0,7-14,0]		[0,0-7,8]	[0,0-7,3]	

*n = Número de participantes; ^†^Md = Mediana; ^‡^[RIQ] = Rango intercuartílico; ^§^p-valor test de Wilcoxon

Para los estudiantes de música, la mediana de la mayoría de las puntuaciones en los signos y síntomas de la escala de ansiedad disminuyeron al finalizar la interpretación en comparación con el inicio, alcanzando diferencias significativas en estado de ánimo ansioso (p=0,038), en insomnio (p=0,024) y en estado de ánimo deprimido (p=0,038). Sin embargo, en la intervención musical en el hospital las puntuaciones fueron muy similares entre al inicio y al final, sin diferencias estadísticamente significativas (p>0,005) y tampoco se encontraron variaciones relevantes al comparar los momentos posteriores al ensayo con los posteriores a la intervención (p>0,005).

Al comparar los dos momentos previos –el inicio del ensayo general y el inicio de la intervención hospitalaria– emergieron diferencias significativas en estado de ánimo ansioso (p=0,024), temor (p=0,008), estado de ánimo deprimido (p=0,038), síntomas somáticos musculares (p=0,031), síntomas somáticos sensoriales (p=0,038), comportamiento en la entrevista (p=0,034) y en la puntuación total de la escala (p=0,012). En esta puntuación global, los niveles de ansiedad fueron significativamente mayores al inicio del ensayo general (11,5; p=0,019) que después del ensayo (5,0; p=0,019), antes de la intervención musical (2,50; p=0,012) y después de la misma (4,0; p= 0,012). Aunque la puntuación en la escala de ansiedad fue ligeramente mayor al finalizar la intervención que al comenzarla, esta diferencia no alcanzó significación estadística (p=0,269) (Tabla 3).

En cuanto a la valoración de la experiencia, el 80% de los estudiantes la calificó como muy buena y 20% como buena, y todos ellos expresaron su disposición a participar nuevamente si se repitiera en cursos posteriores.

## Discusión

Este estudio describe los resultados de una intervención educativa interdisciplinar que integra artes y ciencias de la salud en un entorno sanitario real, orientada al cuidado biopsicosocial y al trabajo en equipo de los estudiantes. Los hallazgos ofrecen una perspectiva novedosa sobre el impacto de la música en el bienestar físico, psicológico y emocional, y subrayan su potencial como complemento terapéutico en el ámbito sanitario.

El aumento significativo de la presión arterial sistólica y de la frecuencia cardíaca tras la intervención, respecto a las mediciones previas, puede atribuirse a una respuesta fisiológica al estímulo musical. Sin embargo, esta variación no es exclusiva de la intervención, pues también resulta de la administración del tratamiento quimioterápico y otros factores. La literatura al respecto se muestra controvertida: algunos estudios en pacientes con cáncer describen una reducciones significativas en parámetros fisiológicos, como la tensión arterial y frecuencia cardiaca, tras intervenciones musicales^(15-16)^, mientras que otros no encuentran diferencias^(17)^.

Al inicio de la intervención musical, los pacientes no manifestaron puntuaciones altas en la escala visual analógica del dolor; por el contrario, refirieron puntuaciones bajas o ausentes, de modo que no se observaron mejoras significativas^(18-19)^, otros estudios tampoco encontraron diferencias^(20-21)^. En cuanto a las puntuaciones en la escala de ansiedad, disminuyeron significativamente tras la intervención. No obstante, dado que la ansiedad puede verse reducida de forma natural después de la administración de quimioterapia, estos resultados deben interpretarse con cautela.

En nuestro estudio, el tamaño del efecto de la música sobre los signos y síntomas de ansiedad fue pequeño. No obstante, estos hallazgos coinciden con otras investigaciones^(15,22-23)^, que se asocian la música en directo con un efecto calmante en comparación con formas pasivas, como la música pregrabada^(17-18,20)^. Una revisión concluyó que las intervenciones musicales pueden reducir la ansiedad, la depresión y el dolor, así como aumentar la esperanza de vida y mejorar la salud fisiológica y psicológica de estos pacientes^(24)^. No obstante, la evidencia disponible sigue siendo limitada y se requieren más estudios para confirmar estos hallazgos^(25)^.

En los estudiantes de música, la significativa disminución de la presión arterial sistólica tras el ensayo puede reflejar una reducción del estrés o la ansiedad anticipatoria previa a la interpretación. Sin embargo, durante la intervención en el hospital no se encontraron cambios significativos en los signos y síntomas de ansiedad, lo que sugiere que el ensayo general ejerce un mayor impacto reductor que la actuación en directo. Aunque la variación en los signos vitales fue mínima al enfrentarse al entorno hospitalario en comparación con los ensayos previos, todos mostraron una tendencia a la disminución, salvo la frecuencia cardíaca, cuyo incremento podría explicarse por el elevado componente emocional de la experiencia. Estos hallazgos, que contrastan con la hipótesis inicial, ponen de manifiesto la controversia existente en la literatura sobre este efecto.

Los signos y síntomas de ansiedad en los estudiantes fueron mayores antes del ensayo y disminuyeron tras la interpretación musical en directo. La ansiedad escénica, común entre los músicos, puede manifestarse desde edades tempranas y afectar negativamente tanto su rendimiento académico como su bienestar profesional^(26-27)^. No obstante, son escasos los estudios que abordan específicamente a estudiantes de música jóvenes^(26)^.

Se resalta la importancia de incorporar, durante la formación musical, estrategias de prevención y autogestión de la ansiedad escénica, aunque la oferta formativa con este objetivo sigue siendo escasa^(27-28)^. Sin embargo, estudios recientes muestran que desde etapas tempranas se están implementando prácticas como el ensayo simulado, el mantenimiento de una actitud positiva, la preparación meticulosa y las técnicas de respiración, con maestros y docentes ejerciendo un papel clave en el desarrollo profesional y emocional de los estudiantes^(26)^.

Este estudio resalta la iniciativa de implicar al estudiantado en situaciones reales complejas que les permitan desarrollar herramientas de adaptación y afrontamiento como parte de su formación. Además, busca fomentar valores de compromiso y responsabilidad frente a la salud y el bienestar mediante el trabajo en equipo interdisciplinar. Paralelamente, el uso de modalidades artísticas, en este caso la música, potencia la empatía, promoviendo la reflexión, la comprensión^(29)^ y la implicación social^(10,30)^. El enfoque en las artes como herramienta educativa constituye, por tanto, un punto de partida para diseñar metodologías docentes que favorezcan el aprendizaje activo.

En este estudio se fomentó la participación de estudiantes de Enfermería en el trabajo de campo, iniciándolos en los pasos elementales de la investigación; las enfermeras ocupan una posición única para identificar las necesidades de atención de pacientes y sus familias y planificar actividades relacionadas con la música. De este modo, la formación temprana en este tipo de intervenciones, en colaboración con disciplinas influyentes en el bienestar, como la música^(10,31)^, es esencial para promover un cuidado holístico del paciente y su entorno^(32)^. Asimismo, este modelo resulta fundamental para impulsar la atención centrada en la persona, facilitar las relaciones y el apoyo entre profesionales, pacientes y familiares o cuidadores, y favorecer el bienestar biopsicosocial y espiritual de los pacientes oncológicos^(33-36)^.

Como estudio piloto, el tamaño de la muestra fue limitado, dado que se desconocía el efecto de la intervención musical y se buscó explorar preliminarmente su viabilidad y posibles tendencias. Este enfoque permite identificar tendencias y ajustar el diseño en futuras investigaciones con muestras más amplias y, por tanto, resulta necesario repetir el estudio en futuros cursos académicos para aumentar el número de participantes y analizar con mayor detalle la relación entre las variables estudiadas y el efecto de la interpretación de música en directo. Además, la intervención en el hospital se vio condicionada por restricciones de tiempo ligadas al calendario académico y por la disponibilidad de los pacientes, lo que redujo la tasa de participación. En consecuencia, incrementar el número de sesiones, añadir un grupo control e implementar un seguimiento longitudinal permitiría obtener resultados más robustos y concluyentes.

A pesar de las limitaciones descritas, la música demuestra ser una herramienta eficaz para reducir la ansiedad en contextos hospitalarios. Para los estudiantes, supone no solo es una disciplina académica, sino también una fuente de bienestar emocional. Este estudio se destaca por su carácter innovador e interdisciplinar, al promover la colaboración entre estudiantes de Enfermería y de Música coordinados por sus responsables académicos, acercándoles a la práctica profesional y enriqueciendo sus currículums académicos. Además, fomenta el trabajo en equipo en un entorno de aprendizaje activo y colaborativo, impulsando la adquisición de habilidades comunicativas, investigadoras y creativas desde los primeros años universitarios^(31)^, lo que facilita la consolidación de futuras colaboraciones.

Para futuras investigaciones, conviene explorar en profundidad qué estilos de música y qué condiciones (duración, entorno, nivel de participación) optimizan los beneficios observados, así como evaluar el impacto prolongado de estas intervenciones musicales. También resulta oportuno implementar programas similares en otros centros e instituciones para comprobar su eficacia en distintos contextos y su influencia en la práctica profesional de los graduados. En el ámbito práctico, integrar la música como terapia complementaria en entornos hospitalarios y educativos, puede mejorar el bienestar de los participantes y promover un enfoque más holístico del cuidado y la educación.

## Conclusión

Las intervenciones de música en vivo pueden contribuir a reducir los signos y síntomas de ansiedad y los parámetros vitales en pacientes con cáncer y estudiantes. En los pacientes, los signos y síntomas de ansiedad, la presión arterial y la frecuencia cardiaca disminuyeron después de la intervención musical. En los estudiantes de música, los signos y síntomas de ansiedad aumentaron en el ensayo general en comparación con la intervención en el hospital. Sin embargo, se necesitan estudios analíticos para confirmar la relación de causa y efecto.

## Data Availability

El conjunto de datos que respalda los resultados de este estudio no está disponible públicamente.
